# Machine learning enables electrical resistivity modeling of printed lines in aerosol jet 3D printing

**DOI:** 10.1038/s41598-024-65693-y

**Published:** 2024-06-25

**Authors:** Mingdong Li, Shuai Yin, Zhixin Liu, Haining Zhang

**Affiliations:** 1 School of Information Engineering, Suzhou University, Suzhou, 234000 China; 2 School of Mechanical and Aerospace, Nanyang Technological University, Singapore, 639798 Singapore

**Keywords:** Printed line electrical resistivity, Aerosol jet printing, Machine learning, Feature extraction, Convolutional neural networks, Mechanical engineering, Materials for devices

## Abstract

Among various non-contact direct ink writing techniques, aerosol jet printing (AJP) stands out due to its distinct advantages, including a more adaptable working distance (2–5 mm) and higher resolution (~ 10 μm). These characteristics make AJP a promising technology for the precise customization of intricate electrical functional devices. However, complex interactions among the machine, process, and materials result in low controllability over the electrical performance of printed lines. This significantly affects the functionality of printed components, thereby limiting the broad applications of AJP. Therefore, a systematic machine learning approach that integrates experimental design, geometrical features extraction, and non-parametric modeling is proposed to achieve printing quality optimization and electrical resistivity prediction for the printed lines in AJP. Specifically, three classical convolutional neural networks (CNNs) architectures are compared for extracting representative features of printed lines, and an optimal operating window is identified to effectively discriminate better line morphology from inferior printed line patterns within the design space. Subsequently, three representative non-parametric machine learning techniques are employed for resistivity modeling. Following that, the modeling performances of the adopted machine learning methods were systematically compared based on four conventional evaluation metrics. Together, these aspects contribute to optimizing the printed line morphology, while simultaneously identifying the optimal resistivity model for accurate predictions in AJP.

## Introduction

Non-contact Direct Ink Writing (NDIW) emerges as an innovative additive manufacturing (AM) technique, enabling the direct creation of customized and flexible electronics from digital files^1–3^. With the ability to directly fabricate features ranging from nanometers to millimeters on both flat and conformal substrates, NDIW proves highly effective in significantly reducing preparation time, material costs, and enhancing feature resolution, which is particularly noteworthy when contrasted with traditional electronics fabrication techniques^4,5^. Furthermore, since NDIW can work with a diverse range of materials, including metals, ceramics, biological materials, carbon nanotubes, and polymers, it has found widespread adoption in the electronic industry, particularly for applications in wearable devices and flexible electronics^6,7^. Among various NDIW techniques, aerosol jet printing (AJP) stands out due to its distinct advantages, including higher resolution (~ 10 μm) and a more adaptable working distance (2–5 mm)^8–10^. This makes AJP a promising technology for the precise customization of intricate electrical functional devices, such as electromagnetic devices^11^, passive components^12^, and compact printed circuit devices^13^. For instance, AJP achieved comparable read distance with significantly thinner antennas (< 1 µm) compared to copper-etched tag (4 µm)^14^. Additionally, AJP system demonstrated its potential for high-volume production by successfully printing a large quantity of collector lines on a solar cell, utilizing a 40 µm nozzle^15^.

While AJP technology presents numerous advantages, intricate interactions among the machine, process, and materials result in low controllability over the electrical performance of printed lines. This significantly affects the functionality of printed components, thereby limiting the broad applications of AJP^16,17^. As the AJP process exerts an indirect yet impactful influence on the electrical resistivity of printed lines via their effect on printed line morphology, researchers tend to optimize AJP process parameters, aiming to enhance the overall electrical performance of printed lines and broaden the applications of AJP. For instance, Mahajan et al. experimentally determined the optimal process parameters for producing high-aspect ratio (thickness-to-width) lines to enhance conductivity^18^. Moreover, Smith et al. and Zhang et al. identified the optimal operating windows to print continuous lines with reduced overspray^19,20^, which is beneficial in preventing short circuits and open circuits in printed components. Additionally, Wang et al. and Zhang et al. utilized statistical modeling in the AJP process for multi-objective optimization of conflicting printed line features^21,22^, aiming to reduce line edge roughness and thereby enhance resistance homogeneity.

Although previous research has qualitatively improved the electrical performance of printed lines, challenges persist due to the absence of quantitative assessments that would enable the accurate adjustment of electrical resistivity through influencing factors^23,24^. For instance, the limited empirical approach cannot ensure high controllability over the homogeneous electrical performance of printed lines^25,26^—a crucial factor for maintaining consistent electrical properties throughout printed components. On the contrary, quantitative analysis is beneficial for achieving the desired electrical resistivity in AJP, which is crucial for precise signal transmission, voltage regulation, and tailored sensor customization across diverse applications. Therefore, quantitative evaluation of electrical resistivity in AJP is indispensable for ensuring high controllability over the printed line electrical performance.

Traditionally, measuring electrical resistivity requires the use of probes and a multimeter, which is a time-consuming process and poses a risk of damaging the surface of printed electronics^27^. Furthermore, this method restricts electrical resistivity assessment to post-printing, hindering real-time adjustments. To address these challenges and achieve desired characteristics efficiently, there is an urgent need to evaluate the electrical resistivity of printed electronics online, consistently, and in a nondestructive manner. Under such circumstances, the assumption is made that the same features of different line samples have a similar impact on the electrical resistivity, and the identification of the main geometrical features of printed lines for quality modeling has gained growing attention in AJP^28,29^. Therefore, a systematic machine learning approach that integrates experimental design, geometrical features extraction, and non-parametric modeling is proposed to achieve printing quality optimization and electrical resistivity prediction for the printed lines in AJP. Specifically, three classical convolutional neural networks (CNNs) architectures are compared for extracting representative features of printed lines, and an optimal operating window is identified to effectively discriminate better line morphology from inferior printed line patterns within the design space. Subsequently, three representative non-parametric machine learning techniques are employed for resistivity modeling. Following that, the modeling performances of the adopted machine learning methods were systematically compared based on four conventional evaluation metrics. Together, these aspects contribute to optimizing the printed line morphology, while simultaneously identifying the optimal resistivity model for accurate predictions in AJP.

The paper is structured as follows. Section “Methodology” introduces the proposed machine learning approach. Then Section "Experimentation" illustrates the experiments. Following that, Section "Results and discussion" covers the feature extraction and modeling results for AJP, with conclusions and future work presented in Section “Conclusions”.

## Methodology

### Introduction of the proposed approach

**Figure 1:**
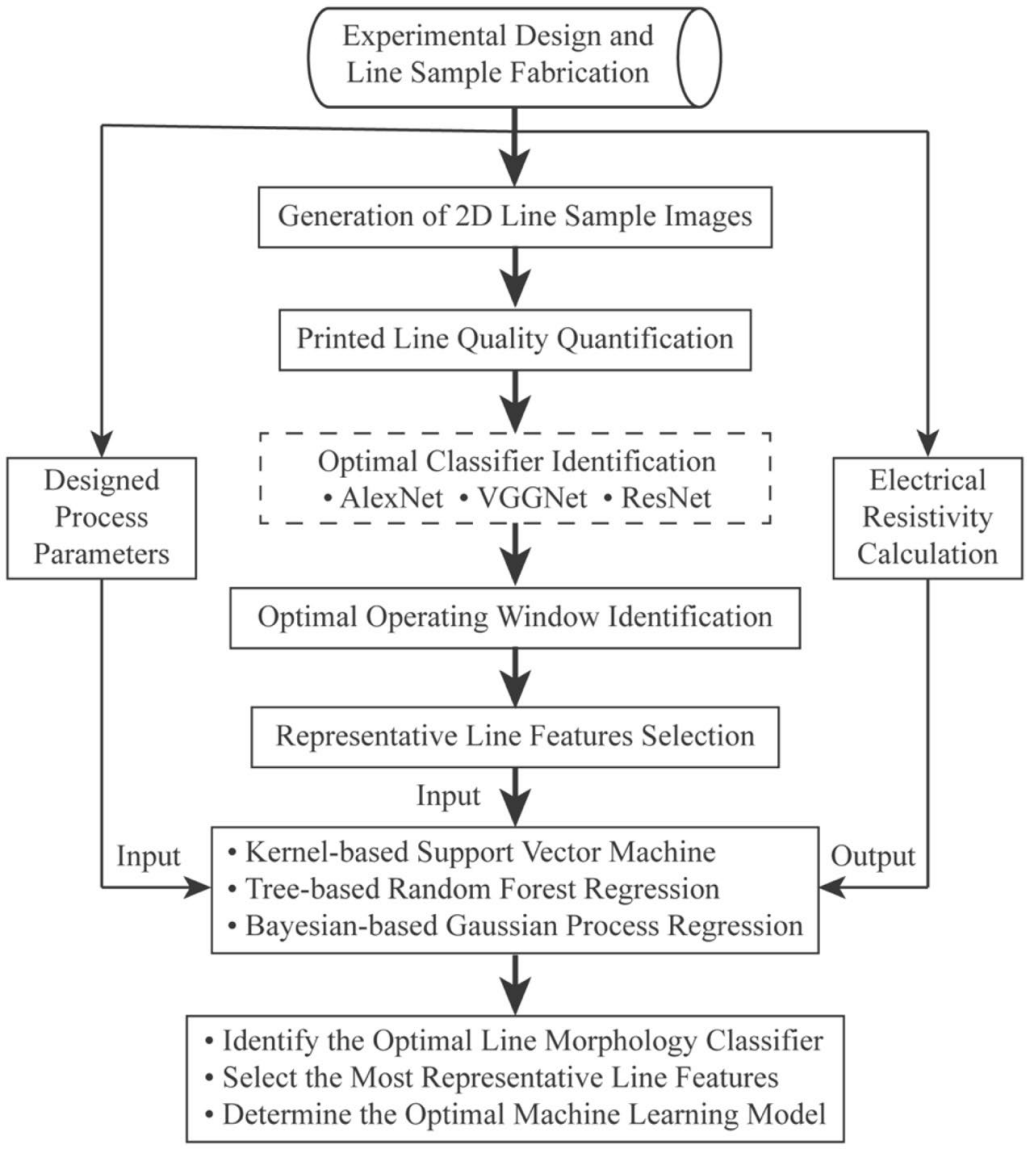
Flowchart of the proposed machine learning approach for electrical resistivity modeling of printed lines in aerosol jet 3D printing.

This research proposes a machine learning workflow for electrical resistivity modeling of printed lines. To assess the effectiveness of the proposed workflow, this study incorporates three convolutional neural network methods and three non-parametric machine learning techniques as case studies. The workflow of the study, along with the methodologies employed, is detailed in Fig. 1. Specifically, line samples with diverse patters are fabricated through designed experiments systematically, resulting in the generation of a 2D image dataset. Subsequently, the printed line quality is quantified based on the adopted printed line characteristics, and three classical CNN architectures are utilized for line morphology classification. The optimal classifier, determined via a comparative analysis of modeling performance among the three CNNs, will extract representative line features and identify an optimal operating window that effectively discriminates inferior printed line patterns from other types within the design space. Following that, the process parameters, encompassing print speed, carrier gas flow rate (CGFR), and sheath gas flow rate (SHGFR) are integrated with the quantified representative line features as model input, with the calculated electrical resistivity of the printed lines serving as the model output. Afterwards, three non-parametric models are adopted for electrical resistivity modeling, leading to the determination of the optimal machine learning model for predicting electrical resistivity in printed lines within the AJP context.

### Convolutional neural networks

**Figure 2:**
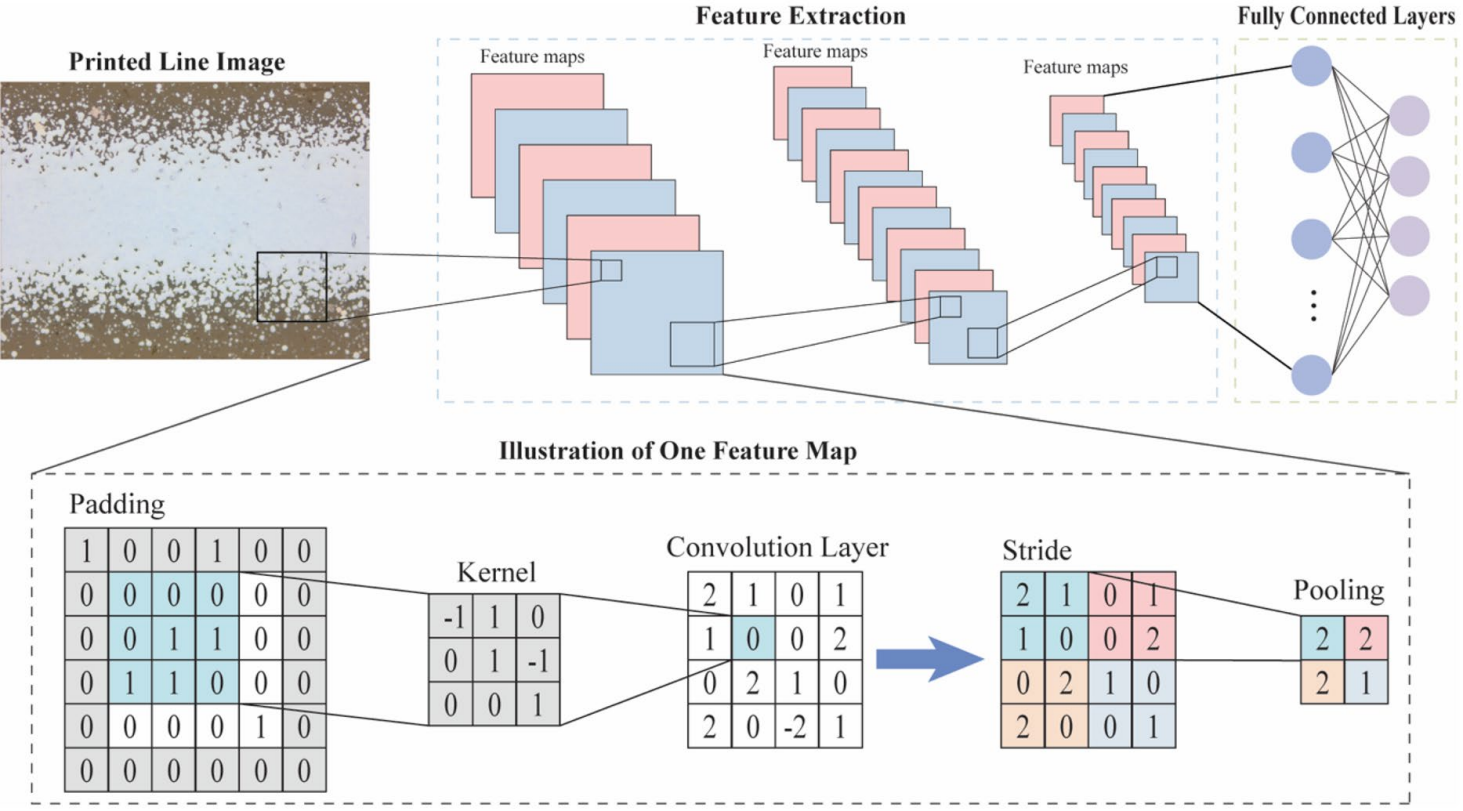
Illustration of a typical CNN architecture.

Convolutional Neural Networks (CNNs) embody a sophisticated class of deep learning architectures meticulously tailored to tackle the complex challenges of image and pattern recognition. Central to the functionality of CNNs is their foundational operational principle: hierarchical feature learning^30^. This fundamental mechanism empowers the network to systematically extract intricate features from visual data, facilitating a nuanced understanding essential for discerning subtle patterns and details with remarkable precision and efficacy. Illustrated in Fig. 2, a standard CNN is comprised of several crucial components: convolutional layers, pooling layers, and fully connected layers. Specifically, the convolutional layers play a crucial role in transferring information from the original image space to a tangible quantity space, systematically extracting local features through convolution operations. Then, pooling layers step in to reduce spatial dimensions while preserving essential details. Following this, the learned features undergo flattening and are seamlessly integrated into fully connected layers, culminating in a holistic comprehension for global pattern recognition. Benefiting from a hierarchical, layered methodology, CNNs can enhance model efficiency through the strategic implementation of weight sharing and parameter sharing. This optimization makes CNNs formidable performers across various applications, particularly excelling in tasks such as object detection, image classification and segmentation across diverse domains^31^. Due to the substantial influence of CNN architecture on performance, this study explores three classical CNN architectures: ResNet, AlexNet, and VGGNet—with the aim of comparing and identifying representative features essential for electrical resistivity modeling. The basic working principles of the adopted CNN architectures are described as follows.

#### AlexNet

The working principles of AlexNet are defined by the incorporation of rectified linear unit (ReLU) activation functions, local response normalization, and dropout mechanisms^32,33^. These components collectively enhance the network's capacity to learn intricate features. Moreover, they play a pivotal role in preventing overfitting during the training process, ensuring the generalizability and robust performance of the developed model across diverse datasets. Specifically, the architecture of AlexNet comprised eight layers, including five convolutional layers succeeded by three fully connected layers.

#### VGGNet

The working principles of VGGNet center around using small 3 × 3 convolutional filters exclusively and stacking multiple layers uniformly^34,35^. Specifically, VGGNet consists of 16 to 19 layers, with each layer featuring 3 × 3 filters, leading to a deeper architecture. This design choice aimed to capture intricate hierarchical features effectively. Unlike AlexNet, VGGNet abandoned the use of local response normalization, relying solely on the stacking of convolutional and pooling layers for feature extraction and spatial downsampling. This architectural simplicity, although computationally more intensive, facilitated a clearer understanding of the network's inner workings.

#### ResNet

ResNet addressed the challenges of training extremely deep networks by introducing residual blocks with shortcut connections^36,37^. These connections allowed the network to skip one or more layers during training, effectively addressing the vanishing gradient problem associated with deep architectures. In contrast to both AlexNet and VGGNet, the design of ResNet prioritized the ease of optimization by focusing on learning residuals, making it more feasible to train networks with a substantial number of layers. The range of ResNet architectures, from 34 to 152 layers, marked a paradigm shift in CNNs, mitigating vanishing gradient issues and enhancing feature representation effectively.

In summary, leveraging the distinctive architectural strengths of AlexNet, VGGNet, and ResNet for extracting printed line features in aerosol jet printing offers significant advantages. Specifically, the deep architecture of AlexNet facilitates the learning of complex hierarchical features, enabling the capture of intricate details in printed lines. On the other hand, the uniform and deep structure of VGGNet can systematically analyze the printed line data layer by layer, facilitating effective feature extraction. Additionally, the residual connections of ResNet allow for the training of deeper architectures, enhancing the accuracy and precision of feature extraction and enabling the extraction of subtle features. Therefore, this research adopts and compares these classical CNN architectures to identify representative features crucial for electrical resistivity modeling.

### Machine learning methods

**Figure 3:**
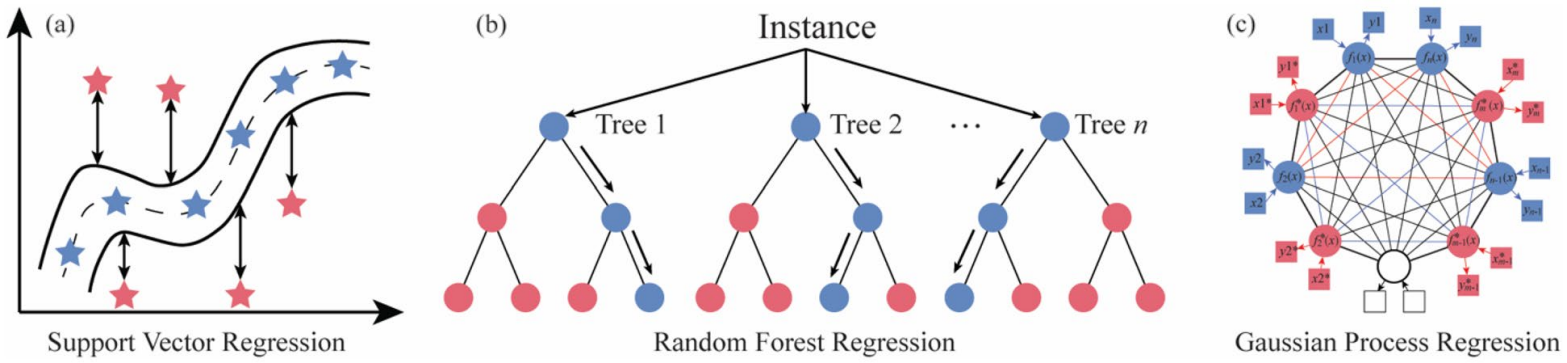
Basic working principles of the adopted non-parametric machine learning methods.

In contrast to parametric machine learning approaches, non-parametric machine learning methods offer the flexibility to fit diverse functions to sampled datasets without assuming specific functional forms or distributions^38,39^. This adaptability enables non-parametric models to achieve modeling performance that is either comparable or superior. In this study, considering the complexity of the AJP process and the absence of prior knowledge for model development, three representative non-parametric methods—kernel-based support vector machine, tree-based random forest regression, and Bayesian-based Gaussian process regression—were chosen for the electrical resistivity modeling of printed lines in AJP. Specifically, this research utilizes representative features extracted from printed lines in each image, along with corresponding process parameters, as input variables. The target response is the printed line resistivity. Figure 3 describes the fundamental working principles of the employed machine learning methods, and the specific illustrations of these algorithms are detailed below:

#### Support vector regression

Support vector regression (SVR) stands as a crucial subset within the support vector machine (SVM) framework, leveraging VC control of the margin, kernels, sparse solutions, and the quantity of support vectors^40^. Despite not enjoying the same level of recognition as SVM, SVR has demonstrated its effectiveness in addressing diverse machine learning challenges within regression problems^41^. As shown in Fig. 3a, by utilizing a training dataset, SVR constructs a regression model expressed as $f\left(x\right)={w}^{T}\phi \left(x\right)+b$, where $\phi \left(x\right)$ denotes the kernel function that originates from the original input space $X$. Here, $w=({w}_{1}, {w}_{2},\ldots {w}_{n})$ denotes the normal vector, and $b$ serves as the intercept. Different from conventional regression approaches, SVR overlooks the absolute predictive error of a training sample if it falls below a specified threshold. Alternatively, exceeding this threshold results in penalties for training points, considering the associated predictive error as a loss function. Consequently, determining the optimal SVR model involves minimizing the overall loss function. Moreover, the robustness of SVR against changes in input space dimensionality provides enhanced efficiency for modeling high-dimensional inputs and will be integrated into future research work of AJP.

#### Random forest

Random Forest (RF) serves as an ensemble machine learning approach, amalgamating the outcomes of multiple decision trees into a unified result. Due to its considerable flexibility, RF is extensively applied in diverse classification and regression scenarios^42,43^. Typically, as shown in Fig. 3b, the RF model development involves four key steps: (1) model training based on random sampling—drawing samples N times with size N from the initial dataset population, each subset is employed to train a decision tree and subsequently reintroduced for resampling; (2) randomly choosing attributes for node splitting—randomly selecting m attributes (where m ≪ M) from M attributes for each sample to split a decision tree node, with the final attribute determined by a specific method; (3) Iteration until Decision Tree Saturation—iteratively executing Step 2 until the decision tree can't be further split, with no pruning; (4) Model Formation—generating a significant number of decision trees based on steps 1–3 to create the RF model. The adaptability and simplicity of RF contribute to its popularity in various machine learning applications.

#### Gaussian process regression

Gaussian Processes Regression (GPR) is a Bayesian-based, probabilistic supervised machine learning framework known for its flexibility and non-parametric modeling capabilities using joint conditional probability, multivariate normal distribution, and kernels^44,45^. Demonstrating comparable or superior accuracy to traditional parametric machine learning methods^46^, GPR is widely adopted for both regression and classification purposes. The construction of a GPR model generally involves three steps: (1) Gaussian prior, (2) joint conditional probability, and (3) posterior marginal distribution. As shown in Fig. 3c, the model treats outputs as latent functions $f(x)$ with a zero-mean Gaussian prior. Then, Bayes posterior inference calculates the joint posterior over $f(x)$, and ${f}^{*}(x)$, incorporating predictive function values at test points. Following that, the posterior predictive distribution for the validation data is derived by integrating out the latent variables from the training set. Given its ability to offer prediction uncertainty^47^, GPR establishes a robust probabilistic framework for diverse machine learning applications.

In summary, GPR models predictions as a distribution of functions, providing probabilistic outputs. RF aggregates predictions from multiple decision trees, while SVR finds the best-fitting hyperplane in a high-dimensional space, allowing for non-linear relationships using kernel tricks.

## Experimentation

### Aerosol jet printing process and experimental setup

In this study, an Optomec® aerosol jet 3D printer equipped with an ultrasonic atomizer was employed for experimental investigations. The standard AJP process involves introducing a liquid functional ink, containing nanoparticle suspensions and solvent, into the atomizer. The ink then undergoes atomization, producing aerosol droplets with diameters ranging from 1 to 5 µm, depending on the specific ink properties. Subsequently, a carrier gas transports the ink aerosol to the printhead, and sheath gas at the nozzle precisely focuses the aerosol, allowing it to exit the nozzle at a velocity of 10–100 m/s. After the printing process, the single pass line samples deposited onto the Kapton® polyimide substrate undergo a sintering step to improve both mechanical and electrical properties. In this research, a Clariant® nanoparticle silver ink (denoted as ink 1), which consists of solvents (water and ethylene glycol), was adopted as the main ink for the experiments.

The process parameters of AJP, which include CGFR, SHGFR, plate temperature, nozzle size, standoff distance, and print speed, play a crucial role in determining printing quality. In this research, we have identified CGFR, SHGFR, and print speed as key influencing parameters that significantly impact the quality of the printed output. This is primarily attributed to the aerodynamic interaction among the carrier gas, annular sheath gas, and aerosol transport, enabling the direct collimation of ink aerosols and the optimization of printed line characteristics. The detailed design space and the corresponding experimental setup of the AJP process are summarized in Table 1, the standard units for CGFR and SHGFR were cubic centimeters per minute (sccm). To fully capture various printed line morphologies for model development, a Latin Hypercube Sampling (LHS) approach is adopted to sufficiently explore the design space^48^, considering the main process parameters such as SHGFR, CGFR, and print speed. This method enables thorough examination of the parameter space, facilitating a comprehensive understanding of the cause-effect correlations between various printed line morphologies and their corresponding electrical resistivity. In this study, a dataset of 4500 images showcasing various printed line patterns was generated from printed line samples taken within the designated design space.

**Table 1:** Design space and the corresponding experimental setup of the AJP process.

Design space			Experimental conditions				
Print speed	CGFR	SHGFR	Standoff distance	Sintering temperature	Ink temperature	Atomization current	Tip size
1–9 mm/s	5–50 sccm	10–100 sccm	3 mm	200 °C	20 °C	0.4 mA	150 μm

### Printed line quality quantification

**Figure 4:**
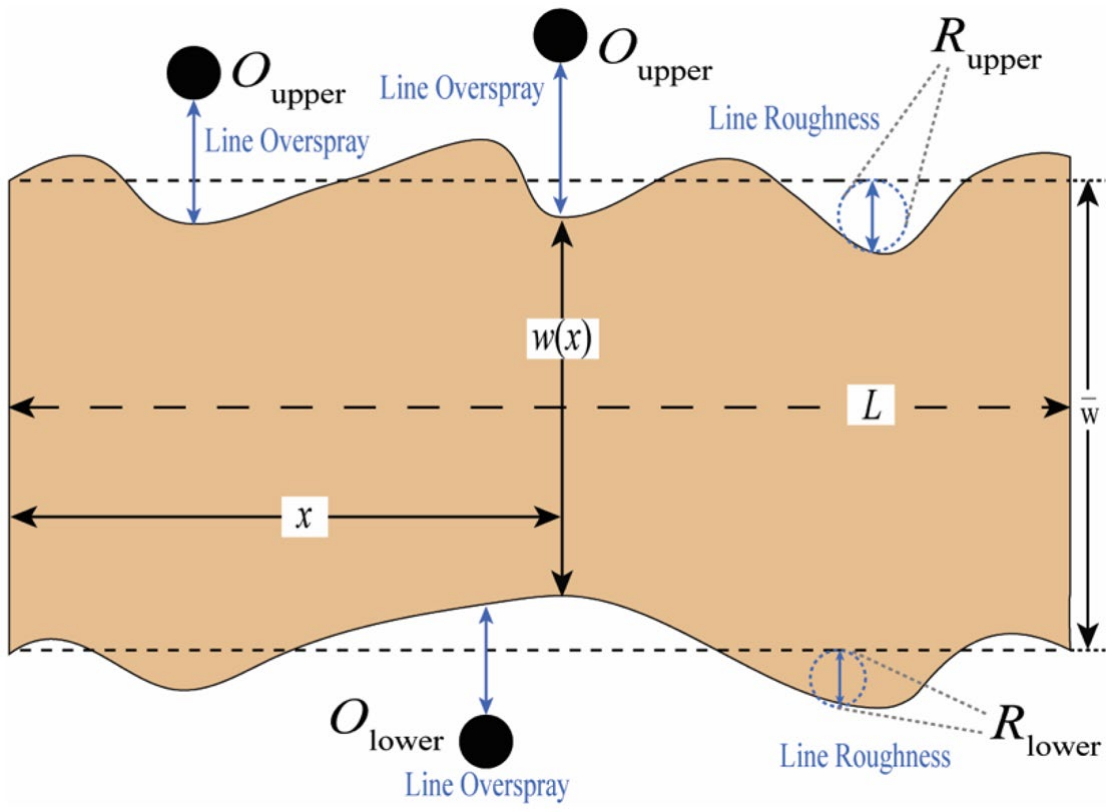
Line features characterization of aerosol jet printing.

As shown in Fig. 4, the printed line quality can be quantified based on the main printed line characteristics, including mean line width ($\overline{w }$), mean line edge roughness (${R}_{m}$), line overspray (${O}_{sp}$), mean line density ($\overline{{L}_{\rho }}$), and line discontinuity (${L}_{disc}$). Specifically, we developed an image processing algorithm to identify the edges of printed lines, and the average width ($\overline{w }$) is determined by analyzing each column of pixels in a discrete manner:

1 $$\overline{w }=\frac{1}{N}\sum_{i=1}^{N}{w}_{i}$$

Then, using the set of mean lines as reference, the mean line edge roughness (${R}_{m}$) and line overspray (${O}_{sp}$) are evaluated in the following manner:

2 $${R}_{m}=\sqrt{\frac{1}{2N}\sum_{i=1}^{N}({R}_{upper,i}^{2}+{R}_{lower,i}^{2})}$$

3 $${O}_{sp}=\frac{1}{2N}\sum_{i=1}^{N}({O}_{upper,i}+{O}_{lower,i})$$

Subsequently, the mean line density ($\overline{{L}_{\rho }}$) is determined by calculating the average of the grayscale intensity values for all pixels located within the identified printed line edges.

4 $$\overline{{L}_{\rho }}=\frac{1}{N}\sum_{i=1}^{N}{I}_{i}-{I}_{b}$$

And the line discontinuity (${L}_{disc}$) refers to the ratio of occurrences when the algorithm misses an edge to the total number of possible defect detection instances.

5 $${L}_{disc}=\frac{{M}_{0}}{2\times N}$$

For the i-th column, ${w}_{i}$ signifies the discretized line width, and N is the total number of all columns. The average intensity in the i-th column is given by ${I}_{i}$, and ${I}_{b}$ is the baseline intensity for the background, applied to offset lighting effects on pixel brightness. Variations in the actual line edge and overspray spots compared to the standard line edge are indicated by ${R}_{i}$ and ${O}_{i}$, respectively.

### Electrical resistivity calculation

**Figure 5:**
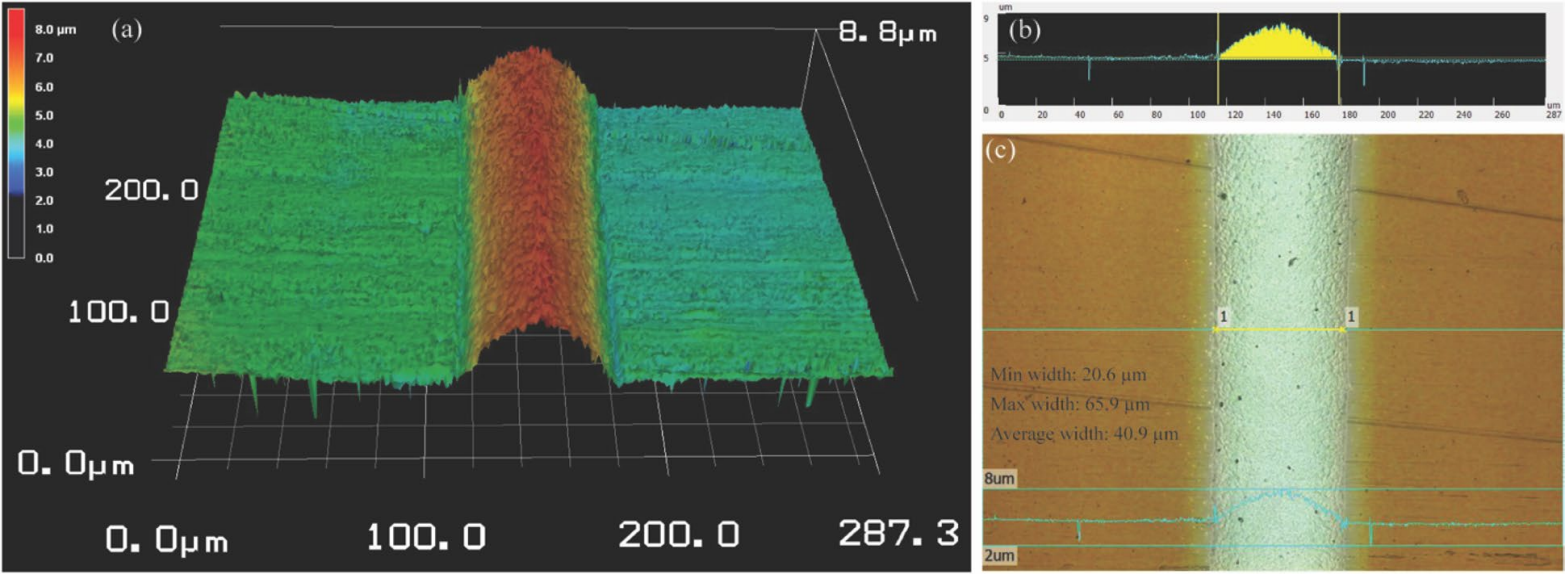
Analysis of 3D profile for resistivity calculation. (**a**) Extracted 3D profile using a confocal laser microscope, (**b**) calculation of cross-sectional area for a deposited line sample, (**c**) the deposited line sample corresponding to (**a**).

Because of the linear correlation between the length and resistance of aerosol jet-printed lines, Ohm's law is applied to elucidate the length-dependent characteristics of resistance in the fabricated line samples.

6 $$R=\rho \frac{L}{S}$$

where the printed line resistance R is determined using the four-point Kelvin resistance measurement technique, with ρ, L, S representing the resistivity, line length and cross-sectional area of the printed line sample, respectively. In this research, three 1.5 cm line samples were arranged linearly on the substrate. The cross-sectional area S for each line sample, depicted in Fig. 5, underwent three measurements, and the values were averaged for subsequent resistivity calculations.

### Model evaluation

Based on the adopted quality index of AJP, the dataset can be labeled into different types and randomly split into six subsets. Five subsets served as the training set, and the remaining subset was used for model testing. The modeling performance of the three classical CNN architectures were evaluated using a cross-entropy loss function ${C}_{EL}$ (training) and prediction accuracy *PA* (testing), facilitating the identification of the optimal CNN classifier for further line feature extraction.

7 $${C}_{EL}=-\frac{1}{n}\sum_{{x}_{j}}\left[{y}_{j}ln{a}_{j}^{L}+\left(1-{y}_{j}\right)ln\left(1-{a}_{j}^{L}\right)\right]$$

8 $$PA=\frac{{N}_{cp}}{{T}_{np}}\times 100\%$$

where ${y}_{j}$ and ${a}_{j}^{L}$ denote the encoded true class label and predicted class label for layer *L*, respectively. ${x}_{j}$ and *n* represent pixel *j* of input image $x$ and the number of training images, respectively. ${T}_{np}$ denotes the total number of predictions. ${N}_{cp}$ represents the number of correct predictions, computed by comparing predicted labels with true labels and tallying the correct predictions. In this research, despite the labels of printed line samples in this study being assigned based on a quantified threshold, it is important to note that the selection of a threshold for labelling different printed line samples may introduce uncertainty, which may potentially impact the classification performance of the developed model.

Due to the potential risk of open circuits caused by discontinuous and sparse lines, as well as the risk of short circuits from excessive overspray and spreading, the developed classifier was further adopted to distinguish these mentioned printed line patterns from the remaining line types, and the identified decision boundary was utilized as the optimal operating window for printing quality optimization. Subsequently, 356 line samples were randomly selected from the identified operating window, and three non-parametric models are employed for electrical resistivity modeling. The calculated resistivity from these samples served as the model output, while the corresponding process parameters were integrated with the identified representative line features as the model input. In this study, four conventional evaluation metrics, R, and R squared (R^2^), root mean-squared error (RMSE) and mean absolute error (MAE), were adopted to comprehensively assess various aspects of the modeling results, despite their lack of independence.

9 $$R=\frac{\sum_{i=1}^{n}(f\left({x}_{i}\right)-\stackrel{-}{f\left({x}_{i}\right)})({y}_{i}-\overline{{y }_{i}})}{\sqrt{\sum_{i=1}^{n}{(f\left({x}_{i}\right)-\stackrel{-}{f({x}_{i})})}^{2}}\sqrt{\sum_{i=1}^{n}{({y}_{i}-\overline{{y }_{i}})}^{2}}}$$

10 $${R}^{2}=1-\frac{\sum_{i=1}^{n}{({y}_{i}-f({x}_{i}))}^{2}}{\sum_{i=1}^{n}{({y}_{i}-\overline{{y }_{i}})}^{2}}$$

11 $$RMSE=\frac{1}{n}\sqrt{\sum_{i=1}^{n}{(f\left({x}_{i}\right)-{y}_{i})}^{2}}$$

12 $$MAE=\frac{1}{n}\sum_{i=1}^{n}|f\left({x}_{i}\right)-{y}_{i}|$$

where $n$ is the number of line samples, ${y}_{i}$ represents the observed actual value, $\overline{y }$ denotes the mean value, $f\left({x}_{i}\right)$ denotes the model prediction with a corresponding mean value of $\stackrel{-}{f\left({x}_{i}\right)}$.

## Results and discussion

### Experimental analysis of the printed line morphology

**Figure 6:**
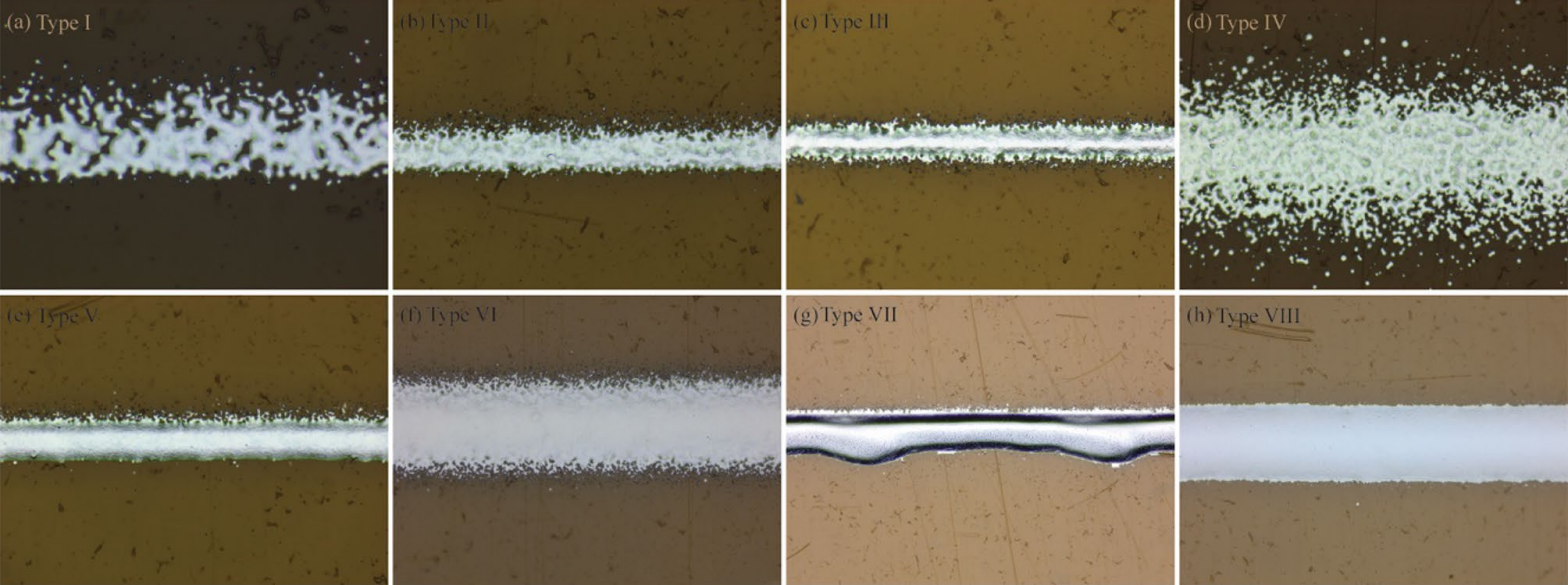
Classification of the printed line morphology in the design space. (**a**) Type I: discontinuous line, (**b**) Type II: sparse line, (**c**) Type III: insufficient thickness line, (**d**) Type IV: high overspray line, (**e**) Type V: high edge roughness line, (**f**) Type VI: low focusing line, (**g**) Type VII: high spreading line, (**h**) Type VIII: normal line.

Based on the adopted quality index of AJP, the printed line samples were labeled into eight types, as depicted in Fig. 6. As the interaction between SHGFR, CGFR, and print speed plays a crucial role in the deposition of line samples with complex features, understanding this interaction is essential to ensure optimal line deposition in AJP processes. Specifically, insufficient atomized ink or a high print speed can lead to the formation of lines with voids and concaves. When the CGFR is low, it fails to transport an adequate amount of atomized ink, resulting in discontinuous lines. To address this, increasing the CGFR beyond a certain point might be necessary. Otherwise, sparse lines and lines that lack sufficient thickness may be produced during the printing process. Conversely, excessive atomized ink or a low print speed can cause the accumulation of nonequilibrium liquid flow within the print channel. This accumulation leads to lines with extensive spreading, which compromises their desired shape and precision. Moreover, unstable aerodynamic interactions between SHGFR and CGFR within the print channel can result in lines with high edge roughness or overspray, further impacting the quality of printed lines. Generally, the printed line roughness ranges from 0.58 to 4.29 μm, while the line overspray spans from 0.15 to 6.56 μm in this research. Therefore, achieving a balanced interaction within the print channel is crucial for ensuring optimal line deposition. This balance effectively addresses issues associated with overspray, spreading, and edge roughness. It involves finding the optimal combination of SHGFR, CGFR, and print speed to ensure sufficient atomized ink delivery while mitigating the accumulation of excessive ink and stabilizing aerodynamic interactions.

**Figure 7:**
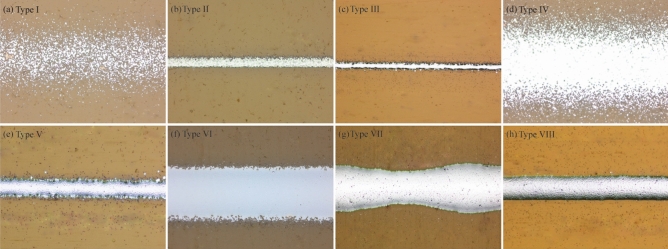
Classification of the printed line morphology in the design space. (**a**) Type I: discontinuous line, (**b**) Type II: sparse line, (**c**) Type III: insufficient thickness line, (**d**) Type IV: high overspray line, (**e**) Type V: high edge roughness line, (**f**) Type VI: low focusing line, (**g**) Type VII: high spreading line, (**h**) Type VIII: normal line.

To further validate the cause-effect relationship between the main process parameters and the printed line characteristics, additional experiments were conducted using Metalon® nanoparticle silver ink (denoted as ink 2). Generally, compared with ink 1, the line samples reprinted using ink 2 demonstrate a similar cause-effect relationship within the designated design space. Specifically, as shown in Fig. 7a and b, the discontinuous or sparse lines are the result of a low CGFR or a high print speed. Moreover, as shown in Fig. 7g, lines printed with excessive CGFR or a low print speed will exhibit extensive spreading. Additionally, Fig. 7c–f illustrate that unstable aerodynamic interactions between SHGFR and CGFR can lead to lines with high edge roughness or overspray. Figure 7h highlights the benefits of achieving a balanced combination of SHGFR, CGFR, and print speed for overall printing quality.

Besides that, the correlations between the printed line characteristics and the corresponding electrical performance were further investigated. As shown in Table 2, the similar characteristics of various line samples printed with different functional inks demonstrate a consistent influence on electrical resistivity. Specifically, insufficient material for deposition results in Type I lines being open circuits, while Type II lines may exhibit high resistivity. Conversely, the electrical conductance of Type IV lines remains relatively low in the design space due to material loss and voids caused by excessive overspray. On the other hand, an improved balance between print speed and CGFR leads to a reduction in resistivity for Type III lines and Type V–Type VI lines within the design space. Although Type VII lines demonstrate better electrical performance, this improved conductivity primarily arises from a disproportionate increase in CGFR. This enlargement of the cross-sectional area for Type VII lines also leads to excessive aerosol accumulation and spreading, resulting in high nonuniformity during printing and nonlinear variations in resistance with length. Compared to the aforementioned line types within the design space, Type VIII lines are more suitable for AJP due to the improved printing quality in terms of electrical performance and line morphology.

**Table 2:** Electrical resistivity of various line samples printed with different functional inks (μΩ cm).

Line type	Type I	Type II	Type III	Type IV	Type V	Type VI	Type VII	Type VIII
Ink 1	$\infty$	7.2–10.6	5.3–6.2	6.7–11.3	2.6–4.9	4.6–5.5	0.9–2.6	1.2–3.5
Ink 2	$\infty$	11.2–13.5	7.6–9.2	10.6–12.1	5.5–7.2	6.9–8.3	3.1–5.6	4.9–6.7

### CNN model development and optimal operating window identification

**Figure 8:**
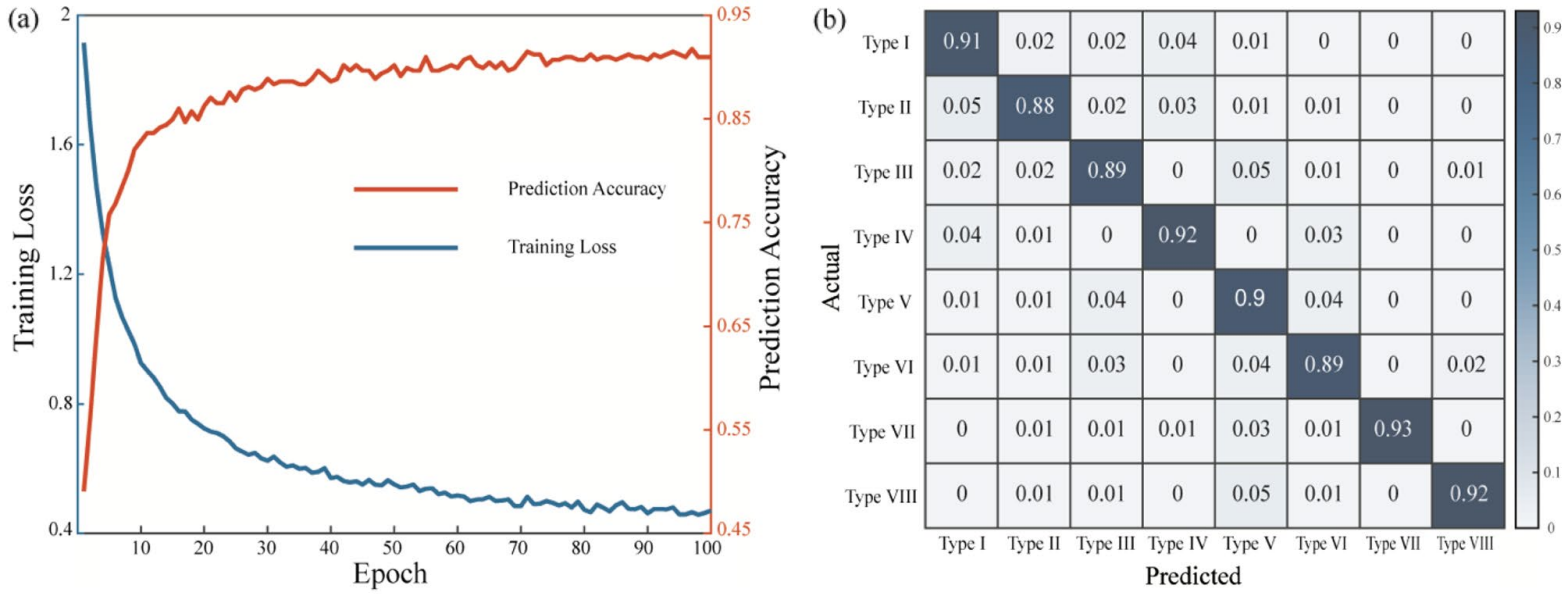
(**a**) ResNet CNN modeling and testing performance for printed line morphology classification, (**b**) testing accuracy matrix with respect to different printed line types on an additional dataset.

Table 3 summarizes the performance of three classical CNN architectures in terms of model training and model testing. Generally, due to architectural differences in the adopted CNN models, ResNet typically exhibits better modeling and testing performance compared to AlexNet and VGGNet. This is attributed to the comparatively shallower architecture of AlexNet, which may limit its ability to discern nuanced features in printed lines. On the other hand, VGGNet, with its uniform architecture and increased depth, excels in learning hierarchical representations but may suffer from computational complexity and low convergence speed. Conversely, the innovative residual connections of ResNet, crucial for capturing intricate morphological details inherent in printed lines, enable it to excel in identifying such features, thereby outperforming AlexNet and VGGNet. In this research, ResNet is determined to be the optimal architecture for representative line feature extraction in this study. Specifically, Fig. 8a presents the evolution of training loss and prediction accuracy with the increasing number of epochs for the ResNet CNN model. Furthermore, to validate the effectiveness of the developed ResNet model, an additional dataset of 600 images was collected under diverse operating conditions using Clariant® functional ink. The obtained testing accuracy matrix, depicted in Fig. 8b, demonstrates the generalizability of the developed model.

**Table 3:** Performance comparison between three classical CNN architectures.

Model	ResNet		AlexNet		VGGNet	
	Run 1	Run 2	Run 1	Run 2	Run 1	Run 2
Training	0.46	0.47	0.42	0.45	0.45	0.49
Testing	91.3%	90.6%	87.6%	89.1%	89.3%	86.5%

**Figure 9:**
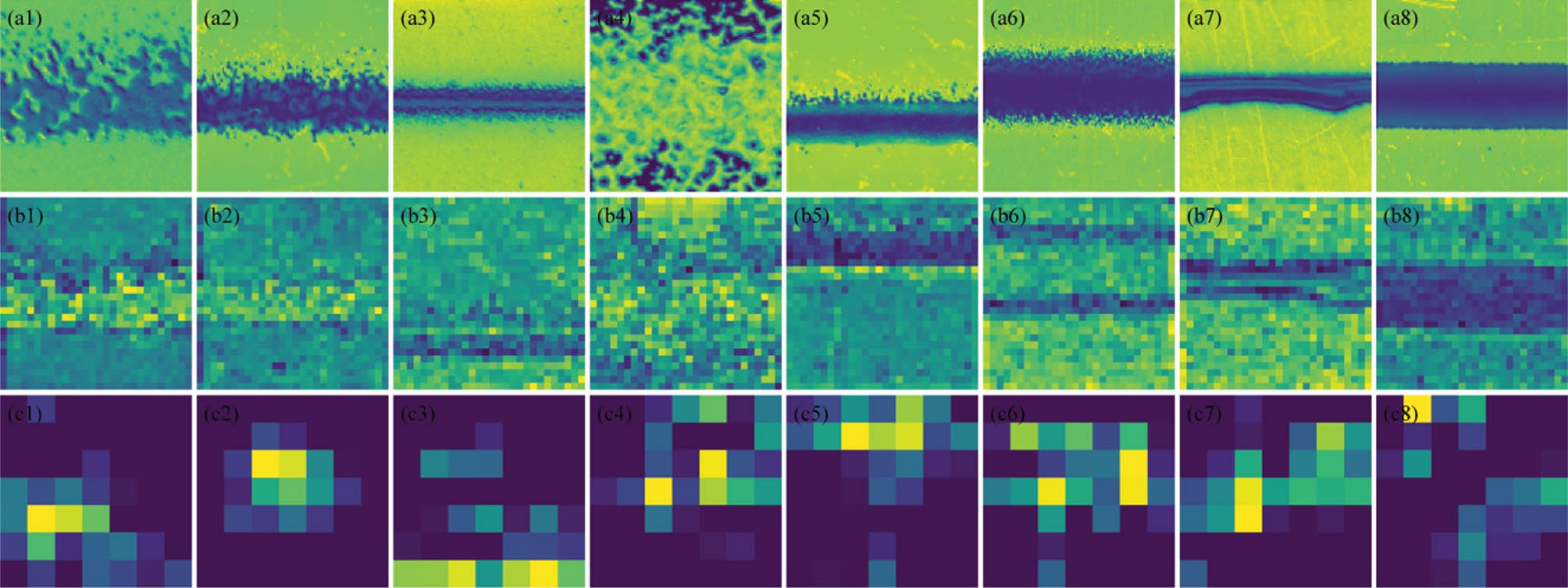
Extraction of geometrical features from printed line samples across different convolutional layers. Features extracted from the (**a1**–**a8**) first convolutional layer, (**b1**–**b8**) fourth convolutional layer, and from the (**c1**–**c8**) last convolutional layer corresponding to line samples in Fig. 6.

Considering the same features of different line samples have a similar impact on electrical performance, the electrical resistivity of printed lines exhibits a strong relationship with geometrical features such as line edge roughness, void size, and void number. Therefore, extracting the geometrical features from printed line samples is essential for characterizing electrical resistivity. In this research, representative line features were extracted using the developed ResNet CNN model, which will be integrated with the corresponding process parameters as input for resistivity modeling. Specifically, Fig. 9 demonstrates the identified features of different printed line patterns based on the developed CNN model. For instance, Fig. 9a1–c1 and a2–c2 extract the deposited voids and concaves within the printed line, potentially resulting in high resistivity. Moreover, Fig. 9a4–c4 highlight excessive overspray of the printed line, contributing to higher electrical resistivity. Conversely, Fig. 9a7–c7 identify the spreading of the line profile, indicating improved conductivity. These findings align with prior domain knowledge. On the other hand, Fig. 9 illustrates examples of extracted geometrical features from various printed line samples across different convolutional layers. Initially, the earliest layer captures the overall morphology of printed lines (Fig. 9a), representing images with minimal processing, encompassing various features redundantly. Conversely, advancement through the convolutional layers reveals a distinct improvement in feature learning. For instance, the third convolutional layer (Fig. 9b) distinguishes specific elements of line samples, such as edges and simple textures, while the final convolutional layer (Fig. 9c) identifies more intricate and abstract textures and patterns. This examination underscores the effectiveness of CNN architecture in extracting relevant information progressively through convolutional layers, while simultaneously filtering out irrelevant elements from images. However, as the interpretability of features in deep convolutional layers is an area of ongoing research, directly interpreting these features, especially those extracted from deeper layers, needs more analysis in future research work^49,50^.

**Figure 10:**
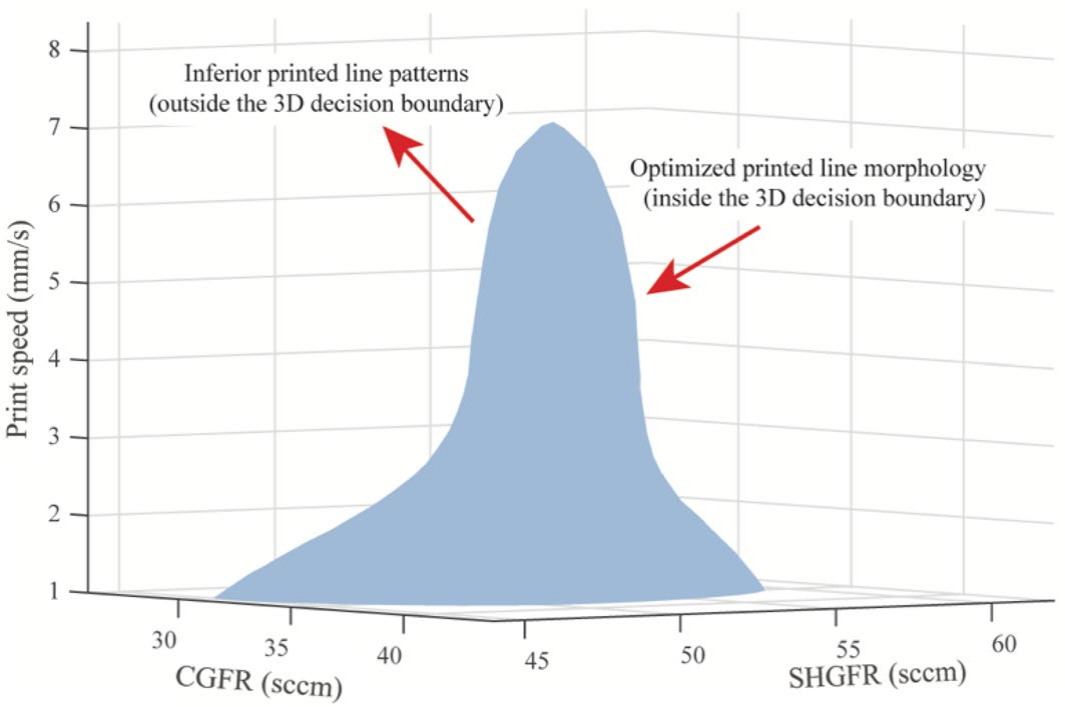
An identified optimal operating window in a 3D design space.

Figure 10 illustrates the identification of a 3D decision boundary, which effectively discriminates inferior printed line patterns (Type I–II, Type IV, and Type VII) from other line types within the design space. The corresponding 3D operating window demonstrates the importance of a balanced interaction between SHGFR, CGFR, and print speed. Specifically, to ensure high-quality printed lines, it is crucial to maintain a balanced aerodynamic interaction between SHGFR and CGFR, while simultaneously controlling the material deposition rate at a reasonable level. This guidance can serve as a fundamental principle for improving printing quality within a 3D design space. However, it should be noted that, unlike printing straight lines, the printed complex geometries (spiral, crossings, etc.) may deform due to varied stress distributions. Therefore, the determined optimal design space may need further modification to alleviate the stress concentration during printing.

### Machine learning based electrical resistivity prediction

**Figure 11:**
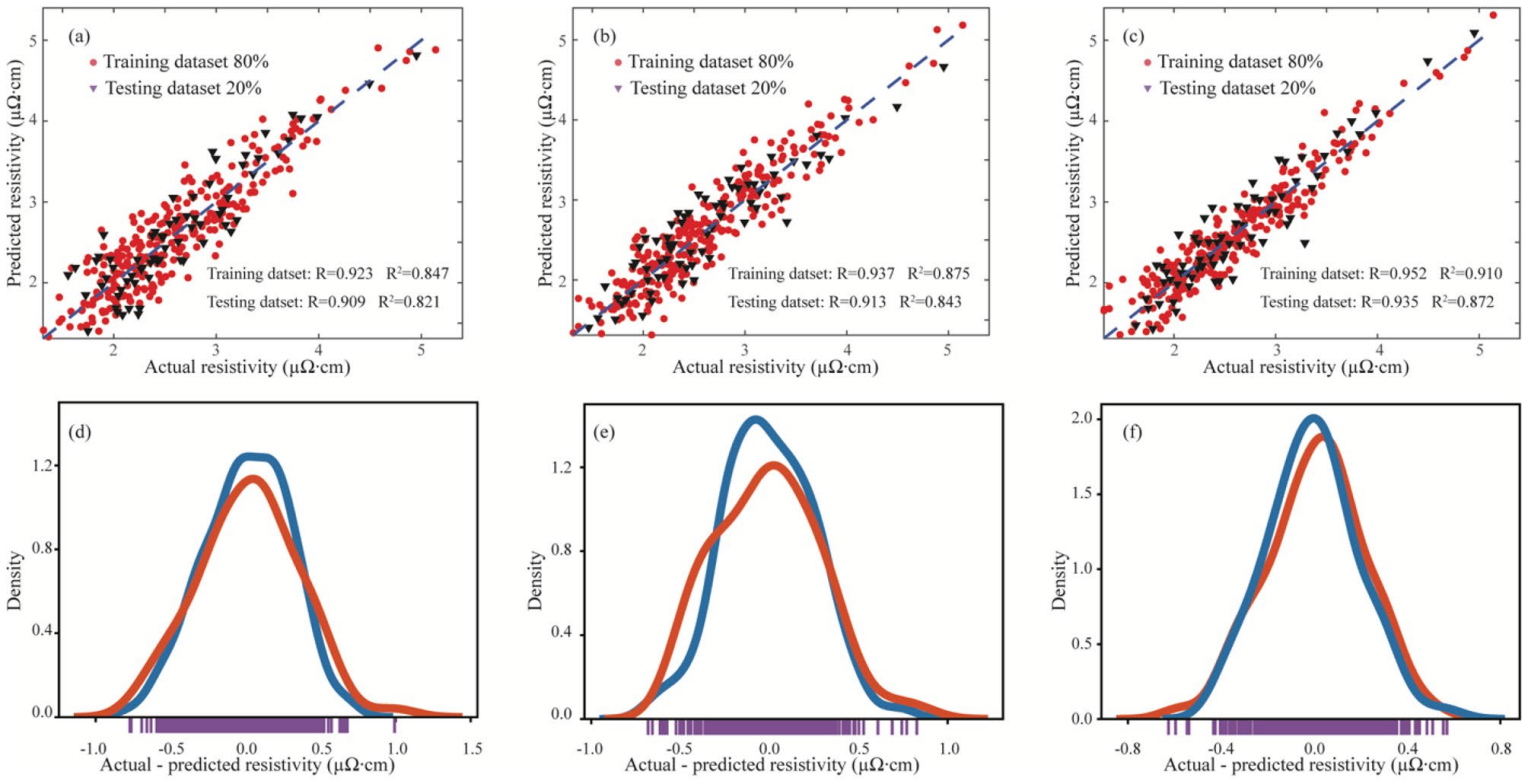
(**a**–**c**) Visualization of the modeling performance of printed line electrical resistivity utilizing RF, SVR, and GPR Models, respectively. (**d**–**f**) Comparison of discrepancies between printed and predicted line electrical resistivity across RF, SVR, and GPR Models, respectively.

The pre-processed dataset underwent division into training and test sets. Specifically, the training set was utilized to develop the model, while the test set assessed its prediction performance. Given the potential impact of dataset split ratio on modeling performance, the test set size ranged from 10 to 45% with a 5% interval, and R determined the optimal split ratio. In this study, the GPR model excelled with a 20% test dataset division, consistent with the modeling performance of RF and SVR. Consequently, an 80%:20% split between training and testing datasets was adopted in this research. Generally, with the increase in CGFR, the electrical performance of the printed line tends to improve. However, this increase also induces significant aerodynamic interactions between SHGFR and CGFR within the printhead, consequently leading to a higher level of nonlinearity in the printing process. As a result, the regression performance decreased for the improved printed line morphology. Specifically, the performance of the developed models, namely RF, SVR, and GPR, was assessed as follows. In Fig. 11a–c, the developed models were compared using R and R^2^, and the obtained values indicate the capability of all three models to effectively capture the overall trend of printed line electrical resistivity. Moreover, Fig. 11d–f provides a visual representation of the disparity and relative frequency of differences between the printed line electrical resistivity and predicted line electrical resistivity. Notably, GPR exhibited better modeling performance, due to its highly adaptable non-parametric modeling technique compared to RF and SVR.

Subsequently, MAE and RMSE were employed to further evaluate the developed models, with the results summarized in Tables 4. In alignment with R and R^2^, the GPR model demonstrated low MAE and RMSE values on both the training and test datasets, confirming its consistent performance. Generally, GPR outperforms Random Forest and SVR due to its probabilistic outputs and flexibility in capturing complex relationships, which enables better modeling of uncertainty and intricate patterns in the collected dataset. Furthermore, to assess the generalizability of the developed GPR model, additional 90 line samples were randomly selected from the collected additional dataset. The resulting values of R, R^2^, RMSE, and MAE were found to be 0.923, 0.859, 0.079, and 0.229, respectively, demonstrating the effectiveness of the developed GPR model for resistivity prediction. In future research work, a smaller operating window can be determined in the design space by increasing the threshold of the entire training dataset, which will further enhance the overall printing quality and the corresponding modeling performance of AJP.

**Table 4:** Modeling performance of printed line width based on four classic evaluation indicators.

Model	Training dataset				Testing dataset			
	R	R^2^	RMSE	MAE	R	R^2^	RMSE	MAE
RFR	0.923	0.847	0.081	0.233	0.909	0.821	0.109	0.265
SVR	0.937	0.875	0.067	0.208	0.913	0.843	0.085	0.237
GPR	0.952	0.910	0.046	0.171	0.935	0.872	0.075	0.217

## Conclusions

In this research, a systematic machine learning approach that integrates experimental design, geometrical features extraction, and non-parametric modeling is proposed to achieve printing quality optimization and electrical resistivity prediction for the printed lines in AJP. Specifically, three classical CNNs architectures are compared for extracting representative features of printed lines, and an optimal operating window is identified to effectively discriminate better line morphology from inferior printed line patterns within the design space. Subsequently, three representative non-parametric machine learning techniques are employed for resistivity modeling. Following that, the modeling performances of the adopted machine learning methods were systematically compared based on four conventional evaluation metrics. Together, these aspects contribute to optimizing the printed line morphology, while simultaneously identifying the optimal resistivity model for accurate predictions in AJP.

Generally, conventional methods often suffer from a lack of quantification and are prone to local optima. In contrast, the proposed machine learning approach offers several distinct advantages. It excels at extracting high-dimensional geometrical features from printed line samples, enabling precise characterization of electrical resistivity, which is crucial for applications such as signal transmission, voltage regulation, and customized sensor development across diverse uses. Moreover, due to its data-driven characteristics, the proposed approach can be applied to other inks, not limited to the specific functional inks used in this study. Additionally, the developed machine learning model has the potential to greatly benefit future model-based optimization. In the future, more influencing factors such as ink properties, standoff distance, tip size, and working distance will be further analyzed^51^. This will lead to the identification of a more robust resistivity model for accurate predictions in AJP.

## Data Availability

The datasets generated and analyzed during the current study are available from the corresponding author on reasonable request.
